# Identification of Proteins Related to Nickel Homeostasis in *Helicobater pylori* by Immobilized Metal Affinity Chromatography and Two-Dimensional Gel Electrophoresis

**DOI:** 10.1155/2008/289490

**Published:** 2007-12-05

**Authors:** Xuesong Sun, Ruiguang Ge, Jen-Fu Chiu, Hongzhe Sun, Qing-Yu He

**Affiliations:** ^1^Institute of Life and Health Engineering, Jinan University, Guangzhou 510632, China; ^2^Department of Chemistry and Open Laboratory of Chemical Biology, The University of Hong Kong, Hong Kong; ^3^Department of Anatomy, The University of Hong Kong, Hong Kong

## Abstract

*Helicobacter pylori (*H. pylori*)* is a widespread human pathogen causing peptic ulcers and chronic gastritis. Maintaining nickel homeostasis is crucial for the establishment of **H. pylori** infection in humans. We used immobilized-nickel affinity chromatography to isolate Ni-related proteins from **H. pylori** cell extracts. Two-dimensional gel electrophoresis and mass spectrometry were employed to separate and identify twenty two Ni-interacting proteins in **H. pylori**. These Ni-interacting proteins can be classified into several general functional categories, including cellular processes (HspA, HspB, TsaA, and NapA), enzymes (Urease, Fumarase, GuaB, Cad, PPase, and DmpI), membrane-associated proteins (OM jhp1427 and HpaA), iron storage protein (Pfr), and hypothetical proteins (HP0271, HP jhp0216, HP jhp0301, HP0721, HP0614, and HP jhp0118). The implication of these proteins in nickel homeostasis is discussed.

## 1. INTRODUCTION

The human gastric pathogen *Helicobacter pylori* (**H. pylori**) is a microaerophilic, spiral-shaped, Gram-negative bacterium responsible for the majority of peptic ulcer diseases in humans [1]. **H. pylori** has been shown to be the causative agent of type B gastritis and peptic ulcerations [2, 3]. Infection with **H. pylori** increases the risks of developing gastric carcinoma and mucosa-associated lymphoid tissue (MALT) lymphoma [4, 5]. Continuously exposed to acidic pH during the process of colonization in the gastric mucus layer, *H. pylori* requires mechanisms to survive acid shocks and to enable growth in such acidic conditions. This bacterium expresses two nickel-containing enzymes: urease and hydrogenase, both of which is important for its colonization. The **H. pylori** urease consists of 12 UreA and 12 UreB, and activation of this apoenzyme requires the insertion of 24 nickel ions [6]. Urease hydrolyzes urea into carbon dioxide and ammonia, thereby neutralizing the nearby environment [7–9]. [Ni-Fe] hydrogen-uptake hydrogenase contains a heterobimetallic center in the large subunit with nickel coordinating to four cysteines, and permits respiratory-based energy production for the bacteria in the mucosa [10, 11]. Therefore, **H. pylori** needs significant amounts of nickel to satisfy its requirement for the maturation of the nickel-containing enzymes [12].

Two kinds of high-affinity, nickel-specific uptake systems are found in **H. pylori**: ATP-binding cassette (ABC)-type transporter (*abc* ABCD) and nickel-cobalt permease (NixA) [13, 14]. However, when excess nickel ions accumulate, they exhibit toxic effects and thus inhibit bacterial growth [15–17]. **H. pylori** has developed a system to maintain nickel homeostasis, keeping a balance between the import, intracellular storage, and export of nickel ions. Analysis of the **H. pylori** genome sequence has discovered several putative ion binders and membrane transporters involved in metal homeostasis [18]. However, the related nickel-interacting proteins have not been fully identified so far.

Immobilized-metal affinity chromatography (IMAC) is a separation technique commonly used for fractionating proteins based on their different binding affinities of the surface-exposed amino acids towards immobilized metal ions [19, 20]. Ni^2+^ belongs to the group of intermediate metal ions, preferring coordination to nitrogen, oxygen, and sulfur, and especially favors binding to the proteins with two exposed vicinal histidines [21]. Elution of the target proteins is achieved by lowering the pH or by adding a competing reagent such as imidazole [22]. Two-dimensional gel electrophoresis (2-DE) is a common choice for separating cellular proteins first by their isoelectric point (p*I*) and then by molecular weight (MW). However, low-abundance proteins may not be detected in 2-DE due to its limited separation capacity. IMAC may compensate this, in some way, by specifically serving to enrich the low-abundance metal-binding proteins and to reduce protein complexity.

In this study, proteomic technology was employed for the first time to isolate and identify candidate proteins involved in nickel transport, storage, and utilization in **H. pylori**, by integrating the powerful tools of Ni-IMAC, 2-DE, and matrix-assisted laser desorption time-of-flight mass spectrometry (MALDI-TOF MS). Those proteins with surface active coordinating residues for binding nickel (and maybe other metals with similar coordinating features) were retained, separated, and analyzed. The information obtained from the identification and functional analysis of these nickel-related proteins may improve our understanding of nickel homeostasis in bacteria.

## 2. MATERIALS AND METHODS

### 2.1. Bacterial culture conditions

**H. pylori** strain 11637 was kindly supplied by Dr. H. H-X. Xia (Department of Medicine, The University of Hong Kong), and cultured in the basal medium, Brucella broth (Difco) with 5% (v/v) fetal bovine serum (FBS; GIBCO/BRL Life Technologies), for 72 hours with orbital shaking (100 rpm) at 37°C in an anaerobic jar with a microaerobic gas-generating kit (Oxoid). Solutions used in this study were prepared with ultra-pure water (18.2 MΩ; Millipore).

### 2.2. Immobilized-nickel affinity

**H. pylori** 11637 cells grown in 100 mL liquid media to mid-log phase were pelleted at 8000× *g* for 5 minutes at 4°C, and washed three times with 10 mL ice-cold phosphate buffered saline (PBS). Cell pellets were resuspended in 10 mL of ice-cold Buffer A (20 mM sodium phosphate buffer, 500 mM NaCl, 10 mM imidazole, 1 mM PMSF, pH 7.4). Bacteria were ruptured with sonication in the presence of 1% v/v Triton X-100. Proteins were recovered (10,000× *g*, 30 minutes), and the supernatant was filtered through a 0.45 *μ*m cellulose acetate syringe filter (Iwaki Glass, Japan) before being loaded onto a house-made, Buffer A-equilibrated Ni-NTA Agarose column (0.5 mL, Qiagen). After washing with 10 column volumes of Buffer A, proteins were eluted with Buffer B (20 mM sodium phosphate buffer, 500 mM NaCl, 500 mM imidazole, pH 7.4). The fractions were concentrated with Centricon YM-3 (Millipore), and buffer-exchanged into rehydration solutions (8 M urea, 4% CHAPS, 1 mM PMSF, 20 mM DTT, 2% pharmalyte pH 3–11) with PlusOne 2-D Clean-up kit (Amersham Biosciences, Buckinghamshire, UK). Protein concentrations were determined by BCA assay (Bio-Rad, Calif, USA) using BSA as the standard. Cell extractswere used immediately or frozen in aliquots at –80°C.

### 2.3. 2-DE

2-DE was carried out with an IPGphor II Isoelectric Focusing (IEF) unit and Hoefer SE 600 Ruby electrophoresis unit (Amersham Biosciences) according to the method detailed elsewhere [23]. Briefly, 100 *μ*g of *H. pylori* protein samples were diluted in rehydration solutions containing traces of bromophenol blue. IEF was carried out with precast 13-cm Immobiline DryStrip (IPG strips; Amersham Biosciences) to generate a nonlinear pH gradient of 3–10. Following IEF, strips were immediately used for the second dimensional SDS-PAGE (10). Proteins were visualized with silver stain, and protein molecular weight (MW) was calibrated with prestained SDS-PAGE marker (Broad range; Bio-Rad). To minimize gel to gel variations, two-dimensional gels were run three times for each sample.

### 2.4. Peptide mass fingerprinting (PMF)

The silver-stained gels were scanned (Image Scanner; Amersham Biosciences) and analyzed with ImageMaster 2D Elite software (Amersham Biosciences) [23]. The normalized intensity (NI) for each protein spot was calculated as the ratio of the spot intensity versus the sum of intensities of the spots present in the whole gel. Protein spots were cut out from the silver-stained gels and enzymatically digested overnight with sequence grade porcine trypsin (Promega) at 37°C. The masses of the digested peptides were determined with a Voyager-DE STR Biospectrometry Workstation (Applied Biosystems, Calif, USA). Protein identification was performed by searching the NCBInr protein database using Protein Prospector (http://prospector.ucsf.edu) (3), with pyroglutamic acid modification of N-terminal glutamine, oxidation of methionine, and protein N-terminal acetylation as permissible modifications. The criteria for searching were set at 50 ppm or better mass accuracy, at least four matching peptide masses, as well as theoretical MW and isoelectric point (p*I*) matching to the estimated values from gels.

## 3. RESULTS AND DISCUSSION

**Figure 1:**
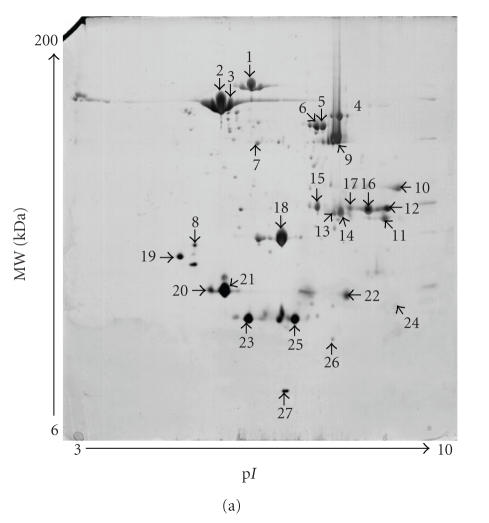

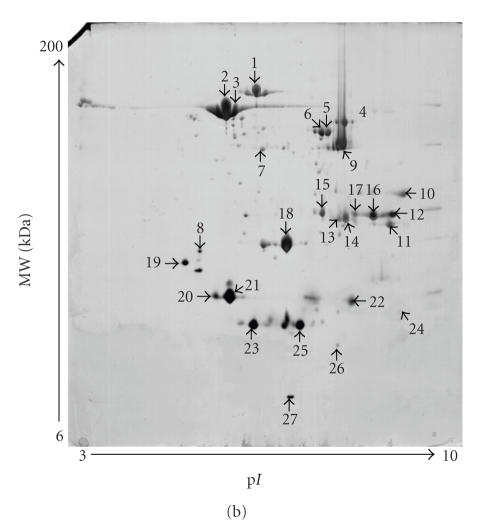
Nickel-interacting proteins in *H. pylori* 11637 analyzed by 2-DE. (a) 2-DE patterns of total Ni-binding proteins in *H. pylori* 11637; (b) 2-DE patterns of *H. pylori* total proteins.

To investigate nickel binding proteins in *H. pylori* under physiologically relevant conditions, a relatively mild and nondenaturing lysis method was applied prior to Nickel-IMAC (Ni-IMAC) loading. Our previous experiments have shown 1-D gels for the total extract of *H. pylori* 11637 cells and extract fractions eluted from a Ni-IMAC column [24], displaying many protein bands for both cases. We have now further separated and identified nickel-interacting proteins in *H. pylori* using 2-DE. The resulting 2-DE images were visualized by silver staining. The gel image of whole cell extracts of *H. pylori* 11637 (see Figure 1(a)) has a protein distribution very similar to the standard proteome pattern of *H. pylori* 26695 [25]. More than 800 protein spots were separated, with molecular weights ranging from 6 to 200 kDa and p*I* values from 3 to 10. Figure 1(a) shows a representative gel image of Ni-related proteins in *H. pylori* 11637. The ratio between the number of Ni-binding proteins and total extracted proteins of whole cell lysates was 1:57, indicating that nickel specifically binds to a restricted number of proteins. Qualitative comparison of 2-D gels between the Ni-enriched eluates (see Figure 1(a)) and the total extracts (see Figure 1(b)) revealed remarkably different spot patterns.

Ni-binding protein spots were excised, trypsin-digested, and subjected to MALDI-TOF MS sequencing. Mass spectra of tryptic peptides were used to identify proteins by matching to the spectra of the protein sequence databases, and the identification was further verified by comparing to the standard proteomic map of *H. pylori* [25]. In total, twenty two proteins were identified as candidate Ni-interacting proteins, as marked in Figure 1(a) and summarized in Table 1. Some of the protein spots could not be annotated due to no substantial MS signals or inadequate peptide coverage for confident identification. Interestingly, five of the proteins identified, including UreA, HspB, fumarase, Pfr, and hypothetical protein jhp0301, show more than one spot in the 2-DE map, indicating the presence of posttranslational modifications. Protein isoforms typically present themselves as a series of spots that differ slightly in their molecular masses and p*I*, that is, numerous closely spaced spots in 2-DE profiles [26–28]. Five proteins (HspA, HspB, fumarase, TsaA, and NapA) were also identified in the Bi-interacting profile [24], indicating that there was some correlation between Bi^3+^ and Ni^2+^ interactions. The Ni-interacting proteins can be classified into five general functional categories: cellular processes (HspA, HspB, TsaA, and NapA), enzymes (Urease, Fumarase, GuaB, Cad, PPase, and DmpI), membrane-associated proteins (OM jhp1427 and HpaA), iron storage protein (Pfr), and hypothetical proteins (HP0271, HP jhp0216, HP jhp0301, HP0721, HP0614, and HP jhp0118).

Nickel homeostasis is required for the establishment of *H. pylori* infection in animals [7], necessitating a balance in the nickel import, storage, and delivery for the synthesis and activity of nickel-dependent metalloenzymes. Proteins involved in nickel homeostasis are potential drug targets. Consequently, an analysis to identify the nickel-interacting proteins in *H. pylori* may be able to reveal candidate proteins for further characterization and validation to provide drug targets.

In order to live in the acidic gastric environment, *H. pylori* continuously synthesize urease catalyzing the hydrolysis of urea to ammonia and carbamate to elevate pH. Urease is an oligomeric Ni-containing heterodimer of UreA and UreB. Both subunits of urease were observed in the 2-DE gel after Ni-NTA enrichment, suggesting each of them can bind nickel ions (see Figure 1(a)). Expression of active urease is essential for *H. pylori* infection in all animal models and for acid survival in vitro [29]. Urease activity is significantly enhanced in the presence of nickel, additional incorporation of Ni^2+^ into the apoenzyme is thus the major regulating event upon higher Ni^2+^ availability.

Heat shock proteins are a group of highly conserved, abundantly expressed proteins with protective advantage through their functions as molecular chaperones, assisting proper folding of a number of substrate proteins that are otherwise destined to aggregation [30, 31]. Both Chaperones HspA and HspB were isolated by Ni-IMAC. HspA and HspB are also involved in urease activation and protection. Urease activity was increased four folds in *E. coli* when coexpressed with HspA, suggesting that HspA possibly plays an important role in the activation of urease, probably by means of its Ni-binding domain in the C-terminus [32]. HspA has two distinct Ni^2+^-binding sites: a high-affinity site (*K*_*d*_ ≈ 2.8 *μ*M) and a lower affinity site (*K*_*d*_ ≈ 30 *μ*M). The *H. pylori* GroEL homologue HspB belongs to the HSP60 family [33], and has been shown to increase the risk of gastric carcinoma, possibly by directly inducing the hyperproliferation of gastric cells [34]. HspB also was suggested to be responsible for the protection and regulation of urease activity [33].

Many identified Ni-interacting proteins in *H. pylori* are involved in antioxidant, antitoxic, and antiinflammation machinery. *H. pylori* TsaA is a major component of the thioredoxin-dependent thiol-specific antioxidant (TSA) system that catalyzes the reduction of hydroperoxides [35] and peroxynitrite [36]. The cleavage of TsaA suppresses the protecting response of *H. pylori* cells against oxidative stress in two possible ways: directly by the way of nucleophilic attacking the peptide bond through metal-bound hydroxide ions, and/or indirectly by way of stimulating the activities of specific proteases. NapA is also directly involved in cell defense in response to oxidative stress. It was named because of its ability to mediate neutrophil adhesion to endothelial cells [37], and to bind to mucin and neutrophil glycosphingolipids [38]. NapA was identified as a 150 kDa DNA-binding dodecamer that protects cells from DNA damage caused by free radicals under oxidative stress [39, 40]. NapA was found to be positively regulated by iron, repressed by ferric uptake regulator (Fur) [40], and unaffected by copper, nickel, or zinc [41]. An excess of iron is potentially harmful as it catalyzes the formation of toxic reactive oxygen species (ROS) via Fenton chemistry. Ferritin protein Pfr, the major iron storage protein of *H. pylori*, is also regulated by iron, nickel, zinc, and copper. The accumulation of this protein under iron-rich conditions allows *H. pylori* to maximize the iron storage capacity in response to an increase in iron availability [44]. It is essential for the bacteria to adapt to low-iron conditions [42–45]. Obviously, Ni-IMAC is also able to trap Pfr with its cation binding affinity.

## 4. CONCLUSION

Immobilized-nickel affinity chromatography and proteomic analysis were integrated to separate and identify the Ni-interacting proteins in *H. pylori*. The results suggest that Ni-interacting proteins are mainly involved in cellular processes, oxidative stress, and metabolism of the bacteria. This study demonstrated that metalloproteomic technique can be utilized to efficiently identify metal-related proteins that may play crucial roles in metal homeostasis. These proteins may be potential targets for designing and constructing drugs to suppress the bacterial infection.

## Figures and Tables

**Table 1:** Summary of nickel-interacting *H. pylori* proteins identified by peptide mass fingerprinting.

Protein no.	Protein name	Identified peptides	% Sequence coverage	MOWSE score	MW (kDa)/p*I*	
					Theor.	Obs.
1*	UreB	21	42	1.595e+10	61.4/5.5	65.5/5.8
2*	60 kDa chaperonin (HspB)	65	75	1.199e+15	58.2/5.5	59.3/5.3
3	60 kDa chaperonin (HspB)	19	37	9.875e+04	58.2/5.5	56.7/5.5
4	Inosine-$5^{\prime }$-monophosphate dehydrogenase (GuaB)	24	51	2.559e+09	51.8/7.7	56.2/8.0
5	Fumarase	22	51	1.097e+11	51.0/6.5	52.6/7.4
6	Fumarase	20	45	1.800e+10	51.0/6.5	52.6/7.0
7	Putative aminotransferase	26	55	2.466e+12	42.4/5.8	40.4/6.2
8	Hypothetical protein HP0271 (fragment)	4	12	980	38.6/5.0	21.1/4.8
9	Cinnnamyl-alcohol dehydrogenase ELI3-2 (Cad)	15	36	1.912e+09	38.6/7.0	40.7/7.8
10	Cell binding factor 2	21	48	5.030e+06	34.0/9.3	31.9/9.8
11	Outer membrane protein jhp1472	11	32	6.291e+04	30.2/9.1	26.7/9.3
12	Hypothetical protein jhp0216	15	57	7.869e+05	29.5/9.1	27.4/9.4
13	Hypothetical protein jhp0301	7	24	1.027e+04	28.6/7.8	27.1/8.0
14	Hypothetical protein jhp0301	19	61	2.461e+05	28.6/7.8	27.1/8.1
15	Putative neuraminyllactose-binding hemagglutinin homolog (HpaA)	13	50	2.381e+05	28.3/7.9	27.0/7.2
16	Urease alpha subunit (UreA)	14	68	1.280e+07	26.5/8.5	27.2/9.0
17	Urease alpha subunit (UreA)	4	88	1.846e+11	26.5/8.5	27.2/8.5
18	Putative alkyl hydroperoxide reductase (TsaA)	12	54	1.297e+07	21.9/5.7	19.9/6.2
19	Inorganic pyrophosphatase (PPase)	4	23	575	14.7/5.0	20.7/4.7
20	Non-heme iron containing ferritin (Pfr)	2	23	369	19.3/5.4	14.4/5.2
21	Non-heme rion containing ferretin (Pfr)	8	56	2.190e+07	19.3/5.4	14.4/5.5
22	Hypothetical protein HP0721	13	44	1.692e+04	17.6/8.9	14.4/8.5
23	Neutrophil activating protein (NapA)	6	48	1.570e+05	16.8/5.7	10.2/6.2
24	Hypothetical protein jhp0118	4	32	1.620e+03	16.6/9.3	12.2/9.8
25	Chaperonin groES (HspA)	6	44	146	13.0/6.1	10.2/6.4
26	Hepothetical protein HP0614	13	56	1.557e+05	13.0/7.0	7.7/8.0
27	4-oxalocrotonate tautomerase (DmpI)	10	85	3.420e+05	7.5/6.0	5.5/6.4

*Confirmed with 2-DE images of *H. pylori* 26695 (http://www.mpiib-berlin.mpg.de/2D-PAGE/EBP-PAGE/index.html).
